# Barriers to mutational testing in patients with gastrointestinal stromal tumors (GIST) – a survey of life raft group members

**DOI:** 10.1186/s12876-022-02548-8

**Published:** 2022-11-15

**Authors:** Denisse Montoya, Jerry W. Call, Jennily Eshak, Pete Knox, Maeven Luedke, Sahibjeet Kaur, Sara Rothschild, Mary Garland, Norman J. Scherzer

**Affiliations:** The Life Raft Group, 155 US 46 Highway, Suite 202, Wayne, NJ 07470 USA

**Keywords:** Gastrointestinal Stomal tumors, GIST, Mutational testing, Biomarker testing, Survey

## Abstract

**Background:**

Due to the low mutational testing rate in patients with Gastrointestinal Stromal Tumors (GIST), The Life Raft Group (LRG), a non-profit organization that provides support, advocacy and conducts research for patients with GIST, analyzed various factors that may have an impact on patients’ ability to receive mutational testing.

**Methods:**

A survey about mutational testing for patients with GIST or their caregivers, was conducted in June 2020. The survey, sent to 1004 GIST patients and caregivers through email, was promoted through social media with instructions to contact the LRG to participate. The survey was designed by the LRG Patient Registry Department. Members of the LRG, regardless of Patient Registry status, were eligible to participate.

**Results:**

A total of 295 patients/caregivers participated in this study (response rate: 29.4%). The percentage of patients who indicated they had received mutational testing was much higher in this survey (80%) than in the general GIST community (26.7%).

Several reasons were cited for having a test, including: “My doctor ordered/suggested that I have it done” (54%); “The Life Raft Group advised/suggested I have it done” (25%); “I asked my doctor to have it done” (22%); “I had it done as part of a clinical trial” (5%); “I am not sure” (3%) and “Other” (14%). Mutational testing resulted in a treatment change in 25% of cases. Patients were able to select more than one option when completing this question resulting in a percentage greater than 100.

**Conclusions:**

The LRG membership is voluntary and proactive; patients who join are more likely to participate in surveys and mutational testing, as well as more likely to have a GIST specialist. Mutational testing can influence understanding of a patient’s GIST and the treatment best suited to each case. These are extremely important findings, as it helps ensure that patients are on the proper treatment, which should lead to better outcomes.

**Supplementary Information:**

The online version contains supplementary material available at 10.1186/s12876-022-02548-8.

## Background

Gastrointestinal Stromal Tumors (GIST) are a rare disease, as per the National Organization of Rare Disorders (NORD), that can occur anywhere along the gastrointestinal tract (GI), but most commonly occur in the stomach and small intestines [1]. When metastases occur, it is usually to the liver or the peritoneum. Approximately half of GISTs are categorized as very low, low, or intermediate risk of recurrence [2] and surgery is typically the only treatment needed for these GISTs. However, the other half of GISTs are high risk or metastatic at diagnosis and typically require additional treatment with tyrosine kinase inhibitors, TKIs, either before or after surgery and in cases where surgery is not possible [3].

Approximately 75–80% of GISTs are driven by mutations in various exons (8, 9, 11, 13, 17) of the *KIT* gene that result in constitutive activation of the KIT receptor [4, 5]. Another 7% are driven by mutations in the *PDGFRA* gene [6]. Apart from some of the very rare *KIT* exon 17 mutations, nearly all the primary *KIT* mutations (exons 8, 9 (may benefit from higher a dose) [7], 11 and 13) respond extremely well to imatinib and about 1/3 of the *PDGFRA* mutations do as well. The other nearly 2/3 of *PDGFRA* mutations that do not respond to imatinib are D842V mutations that occur in exon 18 of *PDGFRA* [6]. These mutations respond to avapritinib, which was approved in 2020 for *PDGFRA* exon 18 mutations including D842V [8]. Other subtypes and mutations in GIST include succinate dehydrogenase (SDH)-deficient GIST and driver mutations in *BRAF, KRAS, NTRK, FGFR1* fusions and other very rare mutations [9–12]. Secondary *KIT* mutations that confer resistance to imatinib can occur in exons 13, 14, 17 and 18 [13, 14]. A total of five different TKIs (imatinib, sunitinib, regorafenib, ripretinib and avapritinib) are currently approved for GIST and they each have different sensitivity profiles against the various mutations [15].

Despite strong guidelines from organizations such as National Comprehensive Cancer Network (NCCN) and College of American Pathologists (CAP) recommending mutational testing, the testing rate for GIST patients in the United States was only 26.7% for patients diagnosed between 2010 and 2015 [16]. Various international guidelines also publish studies on the importance of mutational testing in patients’ treatment, such as British Sarcoma Group (BSG) and European Society for Medical Oncology/European Reference on Rare Adult Solid Cancer (ESMO/EURACAN) [17, 18]. Mutational testing is important not only for the selection of the appropriate treatment in advanced GIST patients, but the results can also help to prevent ineffective treatments from being used in adjuvant settings. A study from Surveillance, Epidemiology, and End Results (SEER) patients, demonstrated that mutational testing has a substantial impact on overall survival (OS) in GIST patients [16]. Due to the beneficial factors of mutational testing, we assessed the barriers that may have an impact on patients’ ability to receive mutational testing.

## Methods

The Life Raft Group is an international, internet-based non-profit patient support, advocacy, and research organization. In June 2020, the LRG conducted a survey of its members regarding mutational testing. The survey was sent to 1004 GIST patients and caregivers through email. The purpose of the survey was to analyze the different factors that may have an impact in obtaining a mutational test among GIST patients. The LRG maintains a large registry of GIST patients and both registry participants and LRG members not in the registry were eligible to participate in the survey. Survey questions were developed by the Patient Registry Department. The contact method was via email and the survey was filled out online using the Qualtrics platform. For some questions, more than one answer could be provided. The data was analyzed with descriptive statistics and frequency tables were compared using Pearson’s chi-squared test. Statistical analysis was performed using Microsoft Excel for Mac version 16.61.1, R version 4.2.1 and RStudio version 2022.07.1. R packages used were, survival version 3.3–1 and R Commander version 2.7–2. The LRG GIST registry was used as a comparison of patient characteristics of survey respondents compared to LRG registry participants. Data comparison from the LRG registry was limited to patients alive (*n* = 1432) at the time of data freeze (8-20-2020).

The survey was divided into two phases. Phase I consisted of questions about demographic information, GIST diagnosis, and treatment. Phase II consisted of questions about how, why, and where mutational testing was performed. The survey questions are included as Table 1.

**Table 1:** Mutation testing survey questions/responses

Demographics	
Questions	Responses
1. Please select the option that best describes you:	□ I’m the Patient □ I’m the Caregiver
**2. Patient’s Gender**: Please select one of the following:	□ Male □ Female
**3. Patient’s Age**: Which of the following best describes your age group?	□ Under 18 □ 19 to 30 □ 31 to 45 □ 46 to 59 □ 60 to 74 □ 75+
**4. Patient’s Location**: Where do you reside? ▼ United States (1) ... Zimbabwe ~ (503)	Country (1) State (2)
Treatments	
5. When were you **diagnosed** with GIST? This information is located on your pathology report. If you do not know the exact date, please provide an estimated date.	(MM/DD/YYYY)
6. Please select the **best option** that describes the primary setting/facility where you received your GIST diagnosis	□ Large hospital or Academic Institution (Teaching hospital with an affiliated medical university) □ Local hospital (small-medium sized hospital) □ Private local doctor/physician or non-hospital based diagnostic center
7. Which of the following best describes your tumor type at diagnosis? Single tumor refers to a tumor in one location; Multifocal tumors are two or more tumors within the same organ; Metastatic tumors or Mets refer to tumors located in different organs.	□ Single Tumor □ Multifocal □ Metastatic (Mets)
8. Did your doctor (who diagnosed you with GIST) provide enough information about your GIST and your treatment plan before prescribing any treatment or testing?	□ Yes □ No □ I don’t remember/ I don’t know
9. Which of the following **best describes** the events taken after your GIST diagnosis? Note: Treatment refers to any chemotherapy medication such as Gleevec, Sutent, Stivarga, etc.	□ Surgery and then started treatment □ Started treatment and then surgery □ Only Surgery □ Only Treatment □ Neither treatment nor surgery
10. What was the date of your surgery? If you do not know the exact date, please provide an estimated date.	(MM/DD/YYYY)
11. When did you start your first treatment? Note: Treatment refers to any chemotherapy medication such as Gleevec, Sutent, Stivarga, etc. If you do not know the exact date, please provide an estimated date.	(MM/DD/YYYY)
12. Did you have progression or recurrence? Note: Progression refers to spread of the disease to a different site and recurrence refers to the tumor(s) returning to the same location after a period of time	□ Yes □ No
13. When did you have your first progression or recurrence? If you do not know the exact date, please provide an estimated date.	(MM/DD/YYYY)
Mutational Testing	
There are different types of testing performed during the journey of GIST patients. One of them is **mutational testing, which is also referred to as biomarker testing**. This test aims to analyze/identify what genes are mutated within that tumor sample. Thus, the results from this test can be used both for diagnosis and for monitoring the success of a targeted therapy. **Example of a mutational result can be: KIT exon 11 p. T574_E583dup** Note: This test is different from pathology testing-which is used to differentiate GIST cells from other cancers by looking at the physiology of the cells. Example of this can be: CKIT positive and DOG1 negative	
14. Have you had **mutational testing** done?	□ Yes □ No □ I do not know
15. Please provide the **date** that you had mutational testing done. If you do not know the exact date, please provide an estimated date.	(MM/DD/YYYY)
16. What were the **results** of your mutational test? Note: A drop-down list with genes commonly mutated in GIST was provided. ▼ BRAF (1) ... I do not know ~ I do not know (23)	Gene (1) Exon (2)
17. Do you have a secondary mutation?	□ Yes □ No
18. What are the results of your secondary mutation? ▼ BRAF (1) ... I do not know ~ I do not know (23)	Gene Exon
**19. Why** was mutational testing done in your case? You can select more than one option	□ I had it done as part of a clinical trial □ My doctor ordered/suggested I have it done □ I asked my doctor to have it done □ The Life Raft Group advised/suggested I have it done □ I am not sure □ Other: Please specify below
20. Did your treatment plan **change** based on your mutational testing results? Note: Treatment refers to any chemotherapy medication such as Gleevec, Sutent, Stivarga, etc.	□ Yes □ No □ I do not know
21. How did your treatment plan change?	□ Switched treatment □ Increased dosage of current treatment □ Decreased dosage of current treatment □ Stopped treatment □ Other: Please specify below
22. What is the name of the facility/lab where the mutational test was performed? You can find this information on the top portion of your mutational report. Examples of facilities/labs: FoundationOne, NIH, OHSU, MSK, Tempus. If you do not know the name of the facility/lab, please write N/A.	Free text field provided
23. Did your doctor explain your mutational testing results?	□ Yes □ No □ I do not know/ I do not remember
24. What is the name of the doctor who recommended/prescribed your mutational test? If the doesn’t apply to your case, please input N/A	Free text field provided
25. What is the name of the institution where your doctor practices? If you do not know the name of the institution or this doesn’t apply to your case, please input N/A Examples of facilities/labs: FoundationOne, NIH, OHSU, MSK, Tempus.	Free text field provided
26. Are you **currently** under the care of the same doctor that prescribed your mutational testing?	□ Yes □ No □ I did not have a doctor that prescribed/recommended mutational testing
27. Why was mutational testing **not done** in your case? You can select more than one option	□ My doctor never mentioned it as a part of my treatment □ My doctor mentioned it but said I did not need it □ Cost/ Insurance □ Not enough tissue □ Mutational testing did not apply in my case (i.e., low risk, metastatic) □ I do not know □ Other: Please specify below
28. Would you be willing to get mutational testing done if applicable in your case?	□ Yes □ No □ I do not know
29. Do you have any comments or remarks that you would like to share with us about your mutational testing experience?	Free text field provided

## Results

### Characteristics of participants

**Fig. 1:**
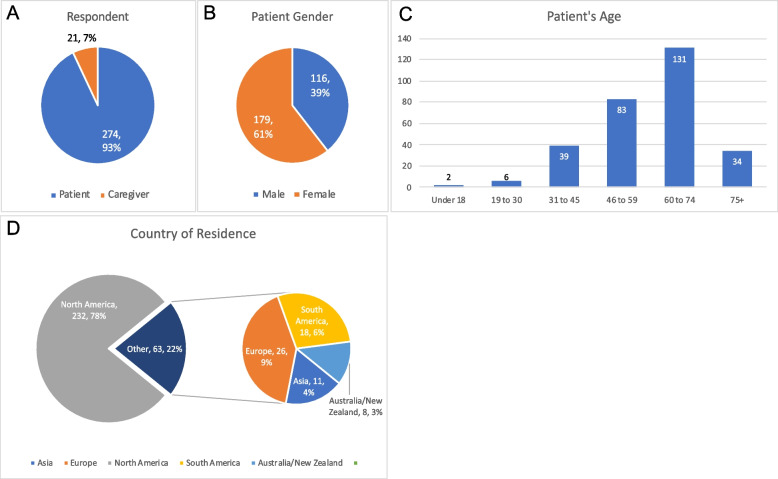
Demographics. **A** - In 93% of cases, the patient was the respondent. **B** - Patient gender was somewhat skewed towards females. **C** - Patient’s age follows a normal distribution for GIST patients. **D** - The majority of respondents were from the United States (78%), which is typical of LRG membership

The majority of survey respondents were patients (*n* = 274, 93%), with 21 caregivers (7%) also participating on behalf of patients, for a total of 295 respondents (Table 2 and Fig. 1A).

**Table 2:** Patient characteristics

	Had Mutation Test?		
	Yes	No	*P* value^a^
All Patients	237 (80.3%)	58 (19.7%)	
**Gender**			
Female	143 (79.9%)	36 (20.1%)	0.81
Male	94 (81.0%)	22 (19.0%)	
**Age distribution**			
< 18	2 (100%)	0	0.74
19 to 30	4 (66.7%)	2 (33.3%)	
31 to 45	34 (87.2%)	5 (12.8%)	
46–59	66 (79.5%)	17 (20.5%)	
60 to 74	103 (78.6%)	28 (21.4%)	
75+	28 (82.4%)	6 (17.6%)	
Unknown			
**Country of Residence**			
North America	183 (78.9%)	49 (21.1%)	0.14
Europe	25 (96.2%)	1 (3.8%)	
South America	14 (77.8%)	4 (22.2%)	
Asia	10 (90.9%)	1 (9.1%)	
Australia/New Zealand	5 (62.5%)	3 (37.5%)	
**Year of Diagnosis**			
< 2000	2 (100%)	0	0.35
2000–2004	26 (81.2%)	6 (18.8%)	
2005–2009	26 (68.4%)	12 (31.6%)	
2010–2014	68 (81.0%)	16 (19%)	
2015–2020	115 (80.3%)	24 (17.3%)	
**Stage at Diagnosis**			
Single tumor	160 (80.4%)	39 (19.6%)	0.10
Multifocal tumor	20 (87.0%)	3 (13.0%)	
Metastatic	57 (78.1%)	16 (21.9%)	

^a^ Pearson’s Chi-squared test comparison of respondents having mutational test versus those without a mutational test

More females responded to the survey than males, (Fig. 1B), 61% female (*n* = 179) and 39% male (*n* = 116). However, a similar female/male ratio (female *n* = 825 (57.6%), males *n* = 607 (42.4%), *p* = 0.33) was observed when only living LRG registry patients are used as a comparison of survey respondents compared to a large GIST population.

Age distribution of survey patient/respondents followed a normal GIST distribution (Fig. 1C), with a peak of respondents aged 60 to 74 (44% *n* = 131). Survey respondents had higher risk than population-based studies which is typical of LRG members with 25% of respondents reporting metastatic disease at diagnosis. Patients from 27 different countries participated, however the majority of patients (78%) were from the United States (Fig. 1D and Supplemental Table 1).

The years of diagnosis for patients responding to the survey were: < 2005, *n* = 34 (12%), 2005–2009, *n* = 38 (13%), 2010–2014, *n* = 84 (28%) and 2015–2020, *n* = 139 (47%).

### Treatments and mutational testing

Patients reported receiving their GIST diagnosis more often in a “large hospital or academic institution (teaching hospital with an affiliated medical university” (*n* = 162, 55%) compared to a “local hospital (small-medium sized hospital” (*n* = 105, 36%) or a “private local doctor/physician or non-hospital based diagnostic center” (*n* = 28, 9%) (Table 3).

**Table 3:** Facilities where patients received their GIST diagnosis

	No.	%
Large hospital or Academic Institution (Teaching hospital with an affiliated medical university)	162	55%
Local hospital (small-medium sized hospital)	105	36%
Private local doctor/physician or non-hospital based diagnostic center	28	9%

In the Mutational Testing sub-section of the survey (Table 1), patients were asked “What is the name of the institution where your doctor practices?” There were 21 institutions listed by three or more patients comprising a total of 117 patients. The most frequently listed sites were: Memorial Sloan Kettering, Dana Farber, Oregon Health Sciences University, MD Anderson, Sylvester Comprehensive Cancer Center and Red de Salud Christus UC (Chile), see Supplemental Table 1.

The percentage of patients with a mutational test was similar (*p* = 0.055) between sites with three or more patients (102 of 118 patients, 86%) and sites with two or less patients (119 of 154 patients, 77%).

These more popular sites had a higher percentage of mutational testing (102 of 118, 86%) compared to sites with two or less patients, with 119 of 154 having a mutational test (77%) and were slightly more likely to explain mutational testing results, 76% versus 69% in the less frequently cited centers.

**Fig. 2:**
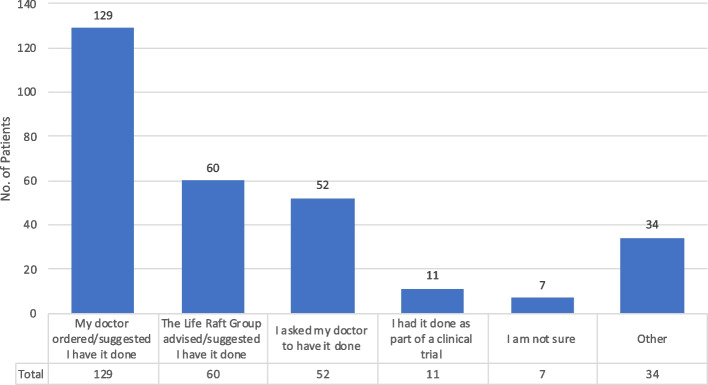
Reasons mutational test was done

This survey identified three major reasons why a mutational test was performed (Supplemental Table 2): The patient’s doctor ordered/suggested the test (54% *n* = 129), the LRG advised/suggested the test (25% *n* = 60) and the patient asked their doctor for the test (22% *n* = 52). In many cases, more than one of these reasons were selected (Table 1-Question 19, Fig. 2).

**Fig. 3:**
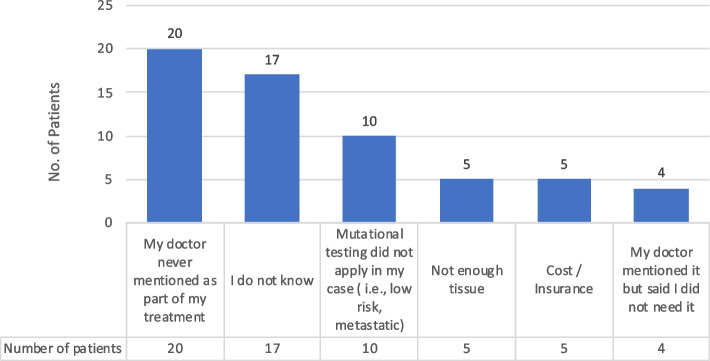
Reasons mutational test not done

Fifty-eight patients with no mutational testing (Table 1-Question 27, Fig. 3) were asked, “Why was mutational testing not done in your case?” Two patients gave multiple responses for a total of 60 responses. The most common two responses were, “My doctor never mentioned it as part of my treatment” (*n* = 20, 33%) and “I do not know” (*n* = 17, 28%). Other reasons included, “Mutational testing did not apply in my case (i.e., low risk, metastatic) (*n* = 10, 17%), “Not enough tissue” (*n* = 5, 8%), “Cost/insurance” (*n* = 4, 7%) and “My doctor mentioned it but said that I did not need it” (*n* = 4, 7%).

### Treatment changes based on mutational testing

**Fig. 4:**
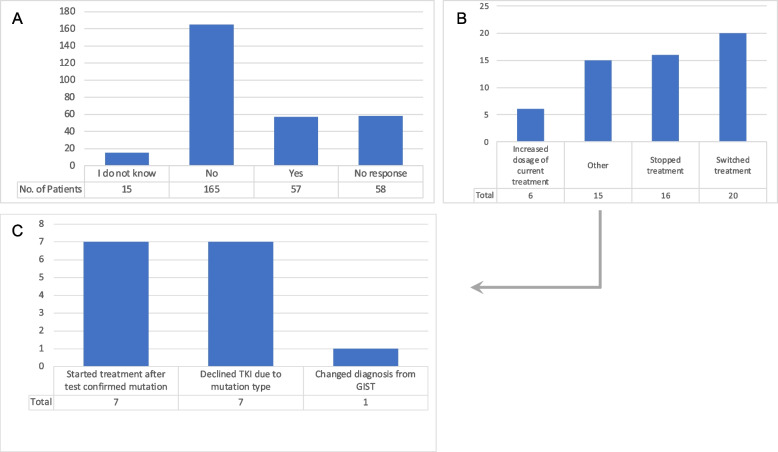
Treatment changes based on mutational testing. **A** – Did treatment change based on mutation test result? **B** – How did your treatment plan change? **C** – Post hoc analysis of “Other” responses from panel **B
**

In this study for 57 of 237 patients (24.5%) with a mutational test, treatment was changed based on the results of the test (Table 1-question 20, Fig. 4). These treatment changes included (Fig. 4B), stopped treatment (*n* = 16, 28%), switched treatment (*n* = 20, 35%), increased dosage of current treatment (*n* = 6, 11%) and other (*n* = 15, 26%). A post hoc analysis of the free text answers from the 15 “Other” responses (Fig. 4C) found that treatment was started for 7 patients (12%) after test confirmed results, 7 patients (12%) declined TKI treatment due to mutation type, 6 patients (11%) switched treatment, and one patient’s (2%) diagnosis was changed from GIST to a different sarcoma (also changing treatment).

## Discussion

A key finding of this study was the critical role that doctors play in whether a patient receives a mutational test. When asked the reason behind why mutational testing was done in their case, 54% of patients reported it was due to the doctor ordering the test or suggesting it be done (Fig. 2), the response with the greatest percentage. This is important because it suggests that reaching out to doctors may have an effect on increasing rates of mutational testing. This is underscored by “My doctor never mentioned it as part of my treatment” being the leading reason (34%) given for why mutational testing was not performed (Fig. 3). Apart from doctors, the next two leading responses for “Why a mutational testing was done?” was that the Life Raft Group suggested having the test done (25%) or the patient asked the doctor themselves (22%) (Fig. 2). This underscores the need for a multi-level approach; in addition to targeting doctors, reaching out to advocacy groups and patients directly may have a beneficial effect as well. Again, this is confirmed by “I do not know” being the second highest reason (29%) (Fig. 3) given as to why a test was not performed, illustrating that an informed patient and/or advocacy group has the power to get a test done, and that an uninformed patient is less likely to succeed in doing so.

While increasing the rate of testing is a worthwhile goal, of more importance is the impact it has on patient outcomes. As mentioned in the previous section, the performance of this test was often quite meaningful in terms of the patient’s treatment. In 25% of the cases, the patient’s treatment was changed based on the results of the mutational testing (Fig. 4A). Even in cases where treatment was not changed, an imatinib-sensitive mutation was often confirmed, offering the GIST patient comfort in an optimized treatment plan. These are both extremely important findings, as it helps ensure that patients are being matched with the proper treatment and leads to better outcomes such as increased survival times [16] and in some cases preventing them from taking ineffective treatments, thus avoiding potentially harmful (and unnecessary) side effects. In addition, studies have shown that receiving early mutational testing has a positive economic impact, as it leads to a more specific prognosis by incorporating the right treatment plan and eliminating avoidable expenses [19]. Mutational testing is a cost-effective approach compared with empirical treatment with imatinib [20].

The percentage of patients receiving a mutational test was significantly higher (*p* = 0.03) for patients living in Europe where 26 of 27 (96.3%) patients reported having a test with other continents varying from 66.7% (South America) to 81.8% (Asia). However, many of these respondents (particularly those from Latin American) had a relationship with the LRG that facilitated mutational testing. Given this selection bias, the percentage of testing from different countries should be interpreted with caution as they may be quite different in the general patient populations of different countries. In particular, many LRG members that are from Latin America obtain mutational testing that is facilitated by the LRG.

Healthcare is, of course, different in different countries/different regions of the world. In the United States cancer patients are more likely to be treated at smaller, local institutions. Larger specialized institutions like academic centers and those with Sarcoma Centers often serve as referral centers. Referrals to these centers are influenced by factors such as geography (distance), case complexity, insurance coverage, access to clinical trials and proactive patients/doctors. In contrast, In Europe most GIST cases are routinely referred to centers with extensive GIST experience and expertise. Latin American and Asia may be similar to Europe (with less certainty and probably more variability from country to country). Although cases numbers are small (Australia = 7, New Zealand = 1), the survey responses from the combined Australia/New Zealand group suggest possible similarities with the United States with patients being diagnosed more frequently in local hospitals versus referral centers. The cost of mutational testing may also affect the availability of testing in some parts of the world. This is unfortunate since mutational testing can actually save money (unpublished LRG analysis) by preventing unnecessary treatment (and side effects) such as adjuvant imatinib for imatinib-insensitive patients (for example patients with D842V mutations in the *PDGFRA* gene).

### Limitations

This study, like all studies, was of course not without its limitations. The Life Raft Group membership has a higher rate of mutational testing than in the general population and also tends to be seen in both local centers and in larger institutions. The patient population in this survey was biased toward proactive patients in two ways. Patients participating in the registry are self-referred/more proactive and patients participating in the survey are further selected for proactive participation. As a result, the percentage of patients reporting having a mutational test in this survey was higher (80% *n* = 237) than in the LRG registry (57% of living patients). Patients in this survey also had a much higher rate of mutational testing than in the general GIST population [15], which was 26.7% of patients diagnosed between 2010 and 2015 in a report of 3888 GIST patients from the Surveillance, Epidemiology, and End Results (SEER) database [15]. Due to these factors, there is an inherent bias in our study population. Only 20% of the respondents did not receive a mutational test (Fig. 3), which is unrepresentative of the general population, particularly in the United States (which were 78% of respondents, Fig. 1). Many patients maintained a relationship with both a local doctor and a GIST/Sarcoma specialty center, in some cases with more than one expert center. When combined with the low percentage of patients in this survey that did not receive a mutational test, it makes any attempt to correlate mutational testing frequency with center size or GIST expertise difficult.

It is reasonable to conclude from this study that both doctors and patients/advocacy groups have a role to play in determining whether a patient receives a mutational test, and if the desire is to increase the rate of testing, then focusing on outreach to these groups could prove beneficial. Also, having looked at responses, it is reasonable to state that mutational testing can have a beneficial role in a patient’s treatment, by either helping reinforce that the selected treatment is the correct one or suggesting a different treatment based on their mutational results, either of which should lead to more favorable patient outcomes. Based on these findings, the recommendation of the authors is to further increase outreach to the aforementioned groups as soon as possible in order to accelerate testing rates and thus allow patients to benefit from these more favorable outcomes.

## Conclusions

In conclusion, mutational testing plays an important role in patients’ treatment. The LRG membership is voluntary and proactive; patients who join are more likely to have an LRG recommended GIST specialist and mutational testing. This shows the role doctors and patient advocacy groups can play in helping increase the rate of mutational testing in GIST patients, which is important because it can positively affect the longevity and quality of life by ensuring that patients are on the proper treatment.

## Supplementary Information

**Additional file 1: Supplemental Table 1.** List of Treatment Centers by Country.

**Additional file 2: Supplemental Table 2.** Why was mutational testing done?.

## Data Availability

Contact the corresponding author for the datasets and other materials used in the survey study.

## Abbreviations

CAP: College of American Pathologists
ESMO: European Society for Medical Oncology
EURACAN: European Reference on Rare Adult Solid Cancer
GI: Gastrointestinal
GIST: Gastrointestinal Stromal Tumors
LRG: The Life Raft Group
NCCN: National Comprehensive Cancer Network
NORD: National Organization for Rare Disorders
SDH: Succinate dehydrogenase
SEER: Surveillance, Epidemiology, and End Results
TKIs: Tyrosine kinase inhibitors
OS: Overall survival
