# Supplementary material for: Barriers to mutational testing in patients with gastrointestinal stromal tumors (GIST) – a survey of life raft group members
Source: BMC Gastroenterol. 2022 Nov 15;22:455. doi: 10.1186/s12876-022-02548-8 (PMC9667594; doi:10.1186/s12876-022-02548-8)
Supplement: Supplementary file 1 — Additional file 1: Supplemental Table 1. List of Treatment Centers by Country. [file 12876_2022_2548_MOESM1_ESM.docx]

**Supplemental Table 1 – List of Treatment Centers by Country**

| **Country/Institution** | **Mutation Test?** | |  |
| --- | --- | --- | --- |
|  | **No** | **Yes** | **Total** |
| **United States** | **42** | **164** | **206** |
| Memorial Sloan Kettering | 1 | 15 | 16 |
| Dana Farber |  | 11 | 11 |
| OHSU | 2 | 7 | 9 |
| MD Anderson |  | 8 | 8 |
| University of Miami Sylvester Cancer Center |  | 6 | 6 |
| Fox Chase Cancer Center | 1 | 5 | 6 |
| Kaiser Permanente | 2 | 4 | 6 |
| Moffitt Cancer Center | 1 | 4 | 5 |
| University of Michigan |  | 5 | 5 |
| Mayo Clinic |  | 5 | 5 |
| Cleveland Clinic |  | 4 | 4 |
| UCLA | 1 | 3 | 4 |
| Stanford |  | 4 | 4 |
| UCSD Moores Cancer Center |  | 3 | 3 |
| Mass General Hospital | 2 | 1 | 3 |
| Johns Hopkins University Hospital | 1 | 2 | 3 |
| Sutter Health | 1 | 2 | 3 |
| Froedtert & Medical College of Wisconsin |  | 3 | 3 |
| Northshore University Health System Kellogg Cancer Center |  | 3 | 3 |
| Centers with 2 respondents (n = 8) | 3 | 13 | 16 |
| Centers with 1 respondent (n = 78) | 23 | 55 | 78 |
| N/A (no details provided) (n = 20) | 7 | 13 | 20 |
|  |  |  |  |
| **Canada** | **1** | **9** | **10** |
| Nova Scotia Cancer Center |  | 2 | 2 |
| Centers with 1 respondent (n = 8) | 1 | 7 | 8 |
|  |  |  |  |
| **Chile** | **2** | **8** | **10** |
| Red de Salud Christus UC |  | 6 | 6 |
| Centers with 1 respondent (n = 8) | 2 | 2 | 4 |
|  |  |  |  |
| **Mexico** | **5** | **3** | **8** |
| IMSS (Mexican Institute of Social Security) | 4 |  | 4 |
| Centers with 1 respondent (n = 4) | 1 | 3 | 4 |
|  |  |  |  |
| **United Kingdom** | **1** | **5** | **6** |
| Centers with 1 respondent (n = 6) | 1 | 5 | 6 |
|  |  |  |  |
| **Australia** | **2** | **4** | **6** |
| Centers with 1 respondent (n = 6) | 3 | 4 | 6 |
|  |  |  |  |
| **Netherlands** |  | **5** | **5** |
| Centers with 1 respondent (n = 5) |  | 5 | 5 |
|  |  |  |  |
| **India** |  | **5** | **5** |
| All India Institute Of Medical Sciences |  | 2 | 2 |
| Centers with 1 respondent (n = 3) |  | 3 | 3 |
|  |  |  |  |
| **Colombia** | **1** | **2** | **3** |
| Centers with 1 respondent (n = 3) | 1 | 2 | 3 |
|  |  |  |  |
| **Italy** |  | **3** | **3** |
| Istituto Nazionale dei Tumori Milan Italy |  | 2 | 2 |
| National Cancer Center in Milan, Italy |  | 1 | 1 |
|  |  |  |  |
| **Argentina** |  | **2** | **2** |
| Centers with 1 respondent (n = 2) |  | 2 | 2 |
|  |  |  |  |
| **Singapore** |  | **2** | **2** |
| Centers with 1 respondent (n = 2) |  | 2 | 2 |
|  |  |  |  |
| **Austria** |  | **2** | **2** |
| Centers with 1 respondent (n = 2) |  | 2 | 2 |
|  |  |  |  |
| **Vietnam** |  | **1** | **1** |
| Centers with 1 respondent (n = 1) |  | 1 | 1 |
|  |  |  |  |
| **Spain** |  | **1** | **1** |
| Centers with 1 respondent (n = 1) |  | 1 | 1 |
|  |  |  |  |
| **Lithuania** |  | **1** | **1** |
| Centers with 1 respondent (n = 1) |  | 1 | 1 |
|  |  |  |  |
| **Sweden** |  | **1** | **1** |
| Centers with 1 respondent (n = 1) |  | 1 | 1 |
|  |  |  |  |
| **Honduras** |  | **1** | **1** |
| Centers with 1 respondent (n = 1) |  | 1 | 1 |
|  |  |  |  |
| **Ukraine** |  | **1** | **1** |
| Centers with 1 respondent (n = 1) |  | 1 | 1 |
|  |  |  |  |
| **Denmark** |  | **1** | **1** |
| Centers with 1 respondent (n = 1) |  | 1 | 1 |
|  |  |  |  |
| **Bolivia** |  | **1** | **1** |
| Centers with 1 respondent (n = 1) |  | 1 | 1 |
| **New Zealand** | **1** |  | **1** |
| Centers with 1 respondent (n = 1) | 1 |  | 1 |
|  |  |  |  |
| **Israel** |  | **1** | **1** |
| Centers with 1 respondent (n = 1) |  | 1 | 1 |
|  |  |  |  |
| **Russia** |  | **1** | **1** |
| Centers with 1 respondent (n = 1) |  | 1 | 1 |
| **Grand Total** | **58** | **237** | **295** |
