# Supplementary material for: Barriers to mutational testing in patients with gastrointestinal stromal tumors (GIST) – a survey of life raft group members
Source: BMC Gastroenterol. 2022 Nov 15;22:455. doi: 10.1186/s12876-022-02548-8 (PMC9667594; doi:10.1186/s12876-022-02548-8)
Supplement: Supplementary file 2 — Additional file 2: Supplemental Table 2. Why was mutational testing done?. [file 12876_2022_2548_MOESM2_ESM.docx]

| **Supplemental Table 2 - Why was mutational testing done?** | |
| --- | --- |
| My doctor ordered/suggested I have it done | 108 |
| (blank) | 58 |
| I asked my doctor to have it done | 26 |
| The Life Raft Group advised/suggested I have it done | 25 |
| I asked my doctor to have it done, The Life Raft Group advised/suggested I have it done | 14 |
| Other | 18 |
| The life raft group advised/ suggested I have it done/ Other | 7 |
| I had it done as part of a clinical trial | 6 |
| My doctor ordered/suggested I have it done, I asked my doctor to have it done | 6 |
| My doctor ordered/suggested I have it done, I asked my doctor to have it done, The Life Raft Group advised/suggested I have it done | 5 |
| My doctor ordered/suggested I have it done, Other | 5 |
| I am not sure | 5 |
| My doctor ordered/suggested I have it done, The Life Raft Group advised/suggested I have it done | 3 |
| I had it done as part of a clinical trial, My doctor ordered/ suggested I have it done; The Life Raft Group advised / suggested I have it done | 2 |
| I had it done as part of a clinical trial, Other | 1 |
| The life raft group advised/ suggested I have it done/ I am not sure | 1 |
| I had it done as part of a clinical trial, My doctor ordered/suggested I have it done | 1 |
| I had it done as part of a clinical trial, The Life Raft Group advised/suggested I have it done | 1 |
| I am not sure, Other | 1 |
| I asked my doctor to have it done, The Life Raft Group advised/suggested I have it done, Other | 1 |
| My doctor ordered/suggested I have it done, The Life Raft Group advised/suggested I have it done, Other | 1 |
| **Total** | **295** |
| **Note:** Some respondents indicated more than one reason for testing. The actual responses are shown in this table. Figure 2 numbers may be higher as the results were aggregated. | |
